# Influence of Ribavirin on Mumps Virus Population Diversity

**DOI:** 10.3390/v13122535

**Published:** 2021-12-17

**Authors:** Mirna Jurković, Anamarija Slović, Dubravko Forčić, Jelena Ivančić-Jelečki, Tanja Košutić-Gulija, Maja Jagušić

**Affiliations:** Centre for Research and Knowledge Transfer in Biotechnology, University of Zagreb, Rockefellerova 10, 10000 Zagreb, Croatia; mirna.jurkovic@unizg.hr (M.J.); aslovic@unizg.hr (A.S.); dforcic@unizg.hr (D.F.); jivancic@unizg.hr (J.I.-J.); tkgulija@unizg.hr (T.K.-G.)

**Keywords:** mumps virus, mutagenesis, virus population diversity, genetic variability, ribavirin

## Abstract

Frequent mumps outbreaks in vaccinated populations and the occurrence of neurological complications (e.g., aseptic meningitis or encephalitis) in patients with mumps indicate the need for the development of more efficient vaccines as well as specific antiviral therapies. RNA viruses are genetically highly heterogeneous populations that exist on the edge of an error threshold, such that additional increases in mutational burden can lead to extinction of the virus population. Deliberate modulation of their natural mutation rate is being exploited as an antiviral strategy and a possibility for rational vaccine design. The aim of this study was to examine the ability of ribavirin, a broad-spectrum antiviral agent, to introduce mutations in the mumps virus (MuV) genome and to investigate if resistance develops during long-term in vitro exposure to ribavirin. An increase in MuV population heterogeneity in the presence of ribavirin has been observed after one passage in cell culture, as well as a bias toward C-to-U and G-to-A transitions, which have previously been defined as ribavirin-related. At higher ribavirin concentration, MuV loses its infectivity during serial passaging and does not recover. At low ribavirin concentration, serial passaging leads to a more significant increase in population diversity and a stronger bias towards ribavirin-related transitions, independently of viral strain or cell culture. In these conditions, the virus retains its initial growth capacity, without development of resistance at a whole-virus population level.

## 1. Introduction

*Mumps orthorubulavirus* (MuV) is a nonsegmented, negative-sense (NNS) RNA virus, belonging to the genus *Orthorubulavirus* in the family *Paramyxoviridae*. It causes a respiratory infection, which, in most cases, naturally passes within two weeks. However, due to the highly neurotropic nature of this virus, aseptic meningitis and encephalitis arise in 1–10% and 0.1% of mumps infections, respectively [1]. An approved antiviral treatment, which would be extremely beneficial in these cases, does not exist. Live-attenuated vaccines are used worldwide and have greatly helped to decrease the incidence rate of mumps. Still, epidemics frequently occur due to genetic differences between vaccine strains and currently circulating viral strains, as well as primary or secondary vaccine failure (reviewed in [2]). Therefore, there is an urgent need to develop more effective and safer MuV vaccines [3].

The MuV genome is a 15,384 nucleotides-long RNA containing seven transcription units that encode nine proteins: nucleo (N), V/phospho/I (V/P/I), matrix (M), fusion (F), small hydrophobic (SH), hemagglutinin-neuraminidase (HN) and large (L) protein [4]. The RNA genome of paramyxoviruses is packaged by the N protein into a nucleocapsid that acts as the template for genome replication and transcription by the RNA-dependent RNA polymerase (RdRp) complex, which consists of the L and P proteins [5]. The L protein has catalytic activity, while the P protein acts as a cofactor that keeps the nucleocapsid in close proximity to the L protein. Since there is no similar enzymatic activity present in the host cells, the RdRp complex represents an attractive target for drug development [6]. Structure-based drug design is, however, hampered by a lack of high-resolution RdRp crystal structures for many RNA viruses, including MuV, and their structural and functional predictions can be made based only on comparison with other related NNS RNA viruses. Regarding L gene, they share six conserved regions (CR-I-CR-VI) and five functional domains: RdRp, poly-ribonucleotidyltransferase (PRNTase), connecting (CD), methyltransferase (MTase), and the C-terminal domain (CTD) [5,7].

RdRp of RNA viruses lacks proofreading activity, thereby generating heterogenous viral populations—termed mutant swarms or quasispecies—in which viral variants cooperate during infection [8,9]. This genetic plasticity allows viruses to evolve and quickly adapt to changing environments. The intrinsic error rate (roughly 10^−4^ mutations per nucleotide copied), or fidelity of RdRp, determines the mutation rate for each virus and the range of genetic variation upon which natural selection can act [8]. Each virus population exists near the maximum mutation rate, called the error threshold, beyond which the quasispecies enters into error catastrophe, losing its genetic information [10].

In recent years, the genetic heterogeneity of viral populations has been exploited for the design and application of antiviral therapeutics and vaccines. Firstly, lethal mutagenesis is being explored as an antiviral approach for driving viral populations toward nonfunctionality through increasing the average mutation rate using mutagens [11,12]. Secondly, it has been demonstrated that the fidelity of RdRp can be manipulated to create less or more diverse viral populations than those that naturally exist, which can result in attenuated phenotype in vitro and in vivo (reviewed in [13]). This can be achieved either by site-directed mutagenesis if the residues involved in RdRp fidelity are known, or by exposing the virus to mutagens such as nucleoside analogs, which allows the selection of viral variants with increased [14,15,16,17] or decreased RdRp fidelity [18,19,20,21]. To our knowledge, so far, there are no data on whether the latter approach can be applied for vaccine design against MuV or to other members of the *Paramyxoviridae* family.

Ribavirin (RBV) is a synthetic nucleoside analog with broad-spectrum antiviral activity that has frequently been used both for the selection of viral variants with changed polymerase fidelity and for exploring the lethal mutagenesis phenomenon. As a guanosine analog, it can be misincorporated into the replicating genomes by RdRp, where it pairs equally well with cytosine (C) and uracil (U), thereby leading to C-to-U and guanine (G)-to-adenine (A) transitions in the progeny genomes [22]. This leads to the induction of error catastrophe as a result of the accumulation of mutations in the viral genome. RBV is reported to have several other direct and indirect mechanisms of action that lead to the inhibition of viral RNA and protein synthesis, including the inhibition of host inosine monophosphate dehydrogenase (IMPDH) and subsequent depletion of GTP pool, the inhibition of viral mRNA polymerase, the inhibition of viral capping, immunomodulatory activity, and the enhanced induction of interferon-related genes (reviewed in [11,12]). Despite having adverse effects, RBV is still recommended (usually in combination with recently introduced directly acting antivirals) for treatment of genotype 1a and 4 hepatitis C virus (HCV) infections, cases of cirrhosis in genotype 3 HCV infections, severe cases of hepatitis E virus infection, severe respiratory syncytial virus pneumonia, and haemorrhagic fevers such as Lassa- and Crimean–Congo-fever [23]. The research on clinical RBV resistance and implication of mutagenesis in vivo has brought conflicting results, and the mechanisms behind it are not fully elucidated. Several studies have identified specific polymorphisms in the NS5B polymerase region of viruses isolated from RBV-treated patients with HCV [24,25,26] or HEV [27], from which only few have been experimentally associated with RBV resistance. Nonetheless, RBV-resistant and sensitive variants recently isolated in the cell culture represent new tools to improve the efficacy of current mutagenic compounds and to identify new compounds with previously unknown antiviral mutagenic activity. Although the antiviral effect of RBV on MuV replication has been previously demonstrated [28,29,30], the molecular mechanism behind it has so far not been elucidated.

In this study, we investigated RBV’s effect on the genetic variability of MuV populations with two goals: (a) to explore if RBV has mutagenic activity against MuV, and (b) to examine if resistance can emerge during in vitro exposure to RBV.

## 2. Materials and Methods

### 2.1. Cells and Reagents

Vero (African Green monkey kidney epithelium) and A549 (human adenocarcinoma) cell lines were obtained from the European Collection of Cell Culture (ECACC, UK Health Security Agency, Salisbury, UK). They were maintained in Minimum Essential Medium with Earle’s salts (MEM) or Dulbecco’s Minimum Essential Medium (DMEM) (both from Capricorn Scientific, Ebsdorfergrund, Germany), respectively, supplemented with 10% foetal bovine serum (FBS) (PAN-Biotech, Aidenbach, Germany), penicillin/streptomycin and L-glutamine (both from Capricorn Scientific, Ebsdorfergrund, Germany) at 37 °C and 5% CO_2_.

The stock solution of RBV (MilliporeSigma, St. Louis, Missouri, USA) was prepared as a 10 mM solution in water for injections, sterile-filtered through a 0.22 μm filter, aliquoted and stored at −20 °C. Working solutions of RBV were prepared in medium containing 2% FBS immediately before use.

### 2.2. Viruses

The MuV laboratory strain (MuVi-C, genotype N) was isolated from the L-Zagreb vaccine (accession number AY685920) through several rounds of plaque purification in Vero cells. MuV strain ZgA/Cro69 (genotype D) was isolated from a child diagnosed with parotitis in 1969 in Zagreb in the amnion of embryonated chicken eggs as one of two viral variants [31]. ZgA/Cro69 virus was further passaged 15× in MRC-5 (normal human lung) cells. Viral titre was determined using a plaque assay, as described in Forcic et al. [32].

All infections were performed in medium containing 2% FBS, penicillin/streptomycin and L-glutamine at 35 °C and 5% CO_2._

### 2.3. Cell Viability Assays

Vero or A549 cells were seeded in 96-well plates at a concentration of 15,000 cells per well in 130 µL of MEM containing 10% FBS or DMEM containing 10% FBS, respectively, and grown overnight. The next day, medium was replaced with 100 µL of fresh control medium containing 2% FBS (untreated cells) or 100 µL of 2-fold serial dilutions of RBV in medium containing 2% FBS (starting from 1000 µM). Cell treatment for each RBV concentration was carried out in six replicates, and wells containing medium only were used as the control. Cells were incubated at 35 °C and 5% CO_2_ for 3 and 4 days for Vero cells and A549 cells, respectively. Next, the medium was replaced with 100 µL of fresh medium containing MTT (3-(4,5-dimethylthiazol-2-yl)-2,5-diphenyltetrazolium bromide) reagent (MilliporeSigma, St. Louis, Missouri, USA) at a final concentration of 0.5 mg/mL, and cells were further incubated for 4 h at 35 °C. The medium was aspirated, cells were then dissolved in 200 µL of DMSO, resuspended, incubated for 5 min at room temperature, and absorbance was read at 570 nm (with reference wavelength of 690 nm) using Multiskan Spectrum (Thermo Fisher Scientific, Waltham, MA, USA).

To distinguish viable and nonviable cells, noninfected cells were seeded and treated with RBV as described in Section 2.6. At indicated time points following treatment, cells were trypsinised and the number of viable cells was determined microscopically in a hemacytometer by trypan blue exclusion.

### 2.4. Kinetics of Viral Replication

Vero or A549 cells were seeded in T-flasks (25 cm^2^) at a concentration of 1.1 × 10^6^ cells in 12 mL of MEM containing 10% FBS or DMEM containing 10% FBS, respectively, and grown overnight. The next day, cell layers were washed twice with phosphate-buffered saline (PBS) and cells were infected at the multiplicity of infection (MOI) indicated in the Results section. After 1 h of adsorption, the virus was removed, cells were washed twice with PBS, and medium containing 2% FBS was added. Cells were further incubated at 35 °C. From day 1 to day 6 after infection, an aliquot was taken from each flask, and stored at −60 °C or below until viral titre was determined.

### 2.5. Effect of Cell Confluence on Viral Replication

Vero or A549 cells were seeded in 12-well plates at concentrations that resulted in approximately 100%, 75% and 50% of cell confluence the next day. Specifically, Vero cells were seeded at concentrations of 1.3 × 10^5^, 1.7 × 10^5^ and 2.1 × 10^5^ cells per well in 1 mL of MEM containing 10% FBS; A549 cells were seeded in 1.1 × 10^5^, 1.5 × 10^5^ and 1.9 × 10^5^ cells per well in 1 mL of DMEM containing 10% FBS. Each concentration was seeded in triplicate. Cells were grown overnight and the medium was removed. Then, cells were washed twice with PBS and infected with viruses at the MOI indicated in the Results section. After 1 h of adsorption, the virus was removed, cells were washed twice with PBS, medium containing 2% FBS was added, and cells were further incubated at 35 °C. Supernatants were collected on day 3 (for Vero cells) or day 4 (for A549 cells) and kept at −60 °C or below until viral titre was determined.

### 2.6. RBV Dose-Dependent Curves

Vero or A549 cells were seeded in 12-well plates at a concentration of 1.7 × 10^5^ cells per well in 1 mL of MEM containing 10% FBS and 1.5 × 10^5^ cells per well in 1 mL of DMEM containing 10% FBS, respectively. Cells were grown overnight; then, the medium was removed and 1 mL of RBV serially diluted in medium containing 2% FBS or medium only was added. Cell treatment for each RBV concentration was carried out in triplicate. Cells were incubated at 35 °C and 5% CO_2_ for 2 h, washed twice with PBS and infected with viruses at the MOI indicated in the Results section. After 1 h of adsorption, the virus was removed, cells were washed twice with PBS, 1 mL of RBV serially diluted in medium containing 2% FBS or medium only was added to cells, and they were further incubated at 35 °C. Supernatants were collected on day 3 (for Vero cells) or day 4 (for A549 cells) following infection and kept at −60 °C or below until viral titre was determined.

### 2.7. Virus Passaging in the Presence of RBV

Vero cells were seeded in 6-well plates at a concentration of 0.45 × 10^6^ cells per well in 2 mL of MEM containing 10% FBS. A549 cells were seeded in T-flasks (25 cm^2^) at a concentration of 1.1 × 10^6^ cells in 12 mL of DMEM containing 10% FBS. Cells were incubated overnight; then, the medium was replaced with the same volume of RBV serially diluted in medium containing 2% FBS or medium only. Cells were incubated at 35 °C and 5% CO_2_ for 2 h, washed twice with PBS and infected with viruses at the MOI indicated in the Results section. After 1 h of adsorption, the virus was removed; cells were washed twice with PBS and further incubated at 35 °C in the medium used for cell pretreatment. Viral titre was determined in the supernatants on day 3 (for Vero cells) or day 4 (for A549 cells) and the next passage was performed with the same MOI used for the first passage.

### 2.8. Next-Generation Sequencing (NGS)

Viral RNA was extracted from 400 µL of cell culture supernatant using the Quick-RNA Viral Kit (Zymo Research, Irvine, CA, USA), following the manufacturer’s recommendations. Isolated RNA was reverse-transcribed with Superscript III (Thermo Fisher Scientific, Waltham, Massachusetts, USA) and amplified using Phusion polymerase (New England Biolabs, Ipswich, MA, USA), according to the manufacturer’s protocol. Primer sequences are shown in Table S1. Fragments were separated on 1% agarose gels, excised, purified using Nucleospin Gel and a PCR Clean-up Kit (Macherey-Nagel, Dueren, Germany) and quantified with a QuantiFluor^®^ ONE dsDNA System (Promega, Madison, WI, USA). Libraries were prepared using an Illumina DNA Prep kit (Illumina, San Diego, CA, USA) following the manufacturer’s protocol. They were quality checked on a 2100 Bioanalyzer using a High Sensitivity DNA Kit (Agilent, Santa Clara, CA, USA). Libraries were pooled and sequenced on a MiniSeq Mid Output Kit (2 × 150 paired-end reads, Illumina, San Diego, CA, USA).

### 2.9. Analysis of Mutational Burden in Viral Populations

The quality of raw reads was assessed with FastQC v0.11.8 and subjected to adapter removal, trimming of bases below a Q-score of 30, and removal of reads shorter than 75 bp using BBDuk within the BBTools package. Paired-end reads were aligned to the reference sequence using Bowtie2 v2.4.2 [33]. Geneious Prime^®^ 2019.2.3 software was then used for majority consensus calling, and reads were realigned to the newly constructed sequence with Bowtie2. Samtools v1.12 [34] was used for further processing of alignments, including filtering reads that had a maximum of two mismatches to the reference sequence. Data regarding the number of obtained reads before and after filtering, as well as mean coverage of alignment, are shown in Table S2. V-Phaser 2 [35] was used to estimate diversity within viral populations. Based on the method described in [36], viral variants present at less than 1% in each sequenced sample were excluded from further analysis using our in-house Python script, which also removes variants found in primer regions. Additionally, the script filters out variants not present in at least five forward and reverse reads. Shannon’s entropy and nucleotide diversity (an average number of nucleotide differences between any two genomes in the sample) [37] were calculated as measurements of viral population diversity. NGS data are available from the NCBI Sequence Read Archive (SRA) database, BioProject ID PRJNA776123.

### 2.10. Statistical Analysis

All graphs were created using GraphPad Prism 9 (Prism Software); 50% inhibitory concentration (IC_50_) values were also calculated using this software. Significant differences among multiple groups were determined using one-way ANOVA and post hoc Tukey–Kramer tests for normally distributed data or Kruskal–Wallis one-way ANOVA when data were not normally distributed. Differences were considered significant for *p* < 0.05.

## 3. Results

### 3.1. MuV Replication in Vero and A549 Cells and Their Sensitivity to RBV

To address the potential mutagenic action of RBV against MuV, we have chosen two different viruses: MuVi-C, a plaque-purified variant obtained from the L-Zagreb vaccine, and ZgA/Cro69, derived from the wild-type virus by continuous passages in the cell culture. As the antiviral activity of RBV is observed through a decrease in viral titre following in vitro infection, we paired chosen MuV strains with substrates where they grow to high titres (Vero cells for MuVi-C; A549 cells for ZgA/Cro69).

**Figure 1:**
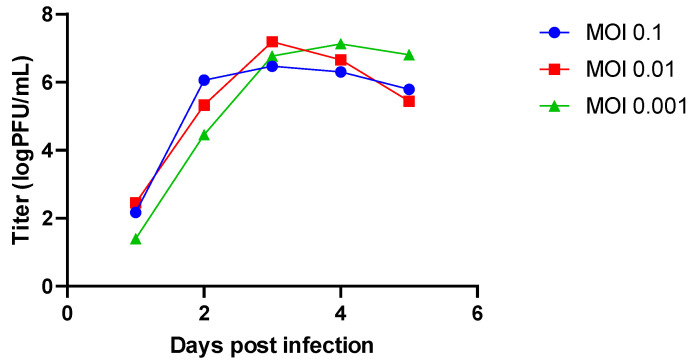
Mumps virus (MuV) replication in Vero cells. Vero cells in layers were infected with MuVi-C at different multiplicities of infection (MOIs). Supernatants were collected from day 1 to day 5 post infection. Titres in the supernatants were determined using a plaque assay.

When infection is carried out at approximately 75% cell confluence, these viruses reach titres higher than 6 log PFU/mL on day three (when using Vero cells and strain MuVi-C) or day four (when using A549 cells and strain ZgA/Cro69) (Figure 1 and Figure S1). Therefore, in the infection experiments with MuVi-C and ZgA/Cro69, we decided to collect supernatants on day three and day four post infection, respectively. In addition, the lowest MOI was chosen in order to minimize the chance of defective viruses being generated, which can arise in mumps-infected cells with only a few passages [38].

We tested the sensitivity of Vero and A549 cells to RBV in our experimental setup (approximately 75% cell confluence, incubation for 3 or 4 days, respectively) using the MTT cell viability assay. The results show that RBV decreased cell viability in a dose-dependent manner with IC_50_ of 399 µM for Vero cells (Figure S2a) and 423 µM for A549 cells (Figure S2b). RBV concentrations higher than 15.62 µM and 31.25 µM led to statistically significant decreases in viability of Vero and A549 cells, respectively. In addition, no cytotoxic effect of RBV was observed in these cells when using the trypan blue exclusion method (data not shown) or by microscopical examination of cells’ layers (Figure S2c,d).

### 3.2. Antiviral Effect of RBV against MuV

**Figure 2:**
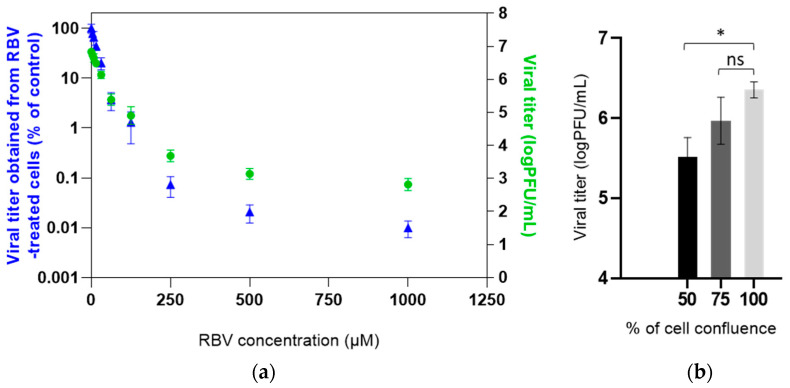
Dose-dependent antiviral effect of RBV against MuVi-C: (**a**) Vero cells were treated with different concentrations of RBV or mock-treated, infected with MuVi-C at MOI 0.001. Then, media with or without RBV were added for an additional 72 h. Titres in supernatants were determined using the plaque assay; titres (logPFU/mL) are plotted on the right y axis, and the titre (number of plaques) of virus obtained from RBV-treated cells as a percentage of the virus titre obtained from untreated, control cells is given on the left y axis. (**b**) Vero cells were seeded to obtain 50%, 75% and 100% confluence on the day of infection, infected with MuVi-C at MOI 0.001. Supernatants were collected after 72 h and titres in supernatants were determined using the plaque assay. Mean and standard deviations are shown from 3 biological replicates. Statistical significance between groups was determined using Kruskal–Wallis one-way ANOVA (* *p* < 0.05; ns, nonsignificant).

We examined the antiviral effect of RBV against MuV by comparing the viral titres in the supernatants of RBV-treated, infected cells versus untreated, infected cells. A549 and Vero cells were pretreated with two-fold serial dilutions of RBV in medium (in a range of 3.9 to 1000 µM) or medium only, infected with MuV and further incubated in the same medium used for pretreatment. A clear dose-dependent antiviral effect of RBV was observed for both viruses (Figure 2a and Figure S3). Fifty percent effective dose (ED_50_) was estimated as 13.67 µM (95% CI 9.526 to 17.82) for MuVi-C (due to fewer RBV concentrations being used, it was not precisely determined for ZgA/Cro69). A flow chart of experiments performed from this point forward is shown in Figure S4.

Next, we addressed the possibility that at RBV concentrations that negatively impact cell viability, a virus replication could have been impaired due to the lower number of cells susceptible to infection and not exclusively because of RBV’s antiviral activity. Infection was performed in cell layers grown at approximately 100%, 75% and 50% confluence. Viral titres in A549 on day four post infection were not affected by the number of cells seeded for infection (Figure S5). Although viral titres on day three post infection were affected by the number of Vero cells available for infection (Figure 2b), this drop in viral titre was at least 500× smaller than the observed drop in titre when RBV was applied. For example, an RBV concentration of 250 µM leads to an almost 50% decrease in cell viability (Figure S2a) and causes 1423× less viral particles; in addition, a 50% difference in the number of cells only results in a 2.5-fold lower viral titre (Figure 2b). Although a contribution of decreased cell viability on RBV’s antiviral effect for concentrations higher than 250 µM cannot be estimated from this experiment, a small additional negative effect that higher RBV concentrations have on the cell viability would not be expected to significantly influence the viral titre.

Taken together, these results confirm RBV’s antiviral activity, independent of strain used in our experimental system, and suggest that the number of cells available for infection makes only a minor contribution to the overall RBV’s antiviral effect.

### 3.3. RBV Effect on Genetic Diversity of MuV Populations after Only One Passage

To estimate if RBV activity includes the induction of mutations in the MuV genome, MuVi-C and ZgA/Cro69 viruses passaged only once in the medium containing 250 µM RBV or control medium, in two independent experiments, were sequenced using NGS. Due to the low viral titre of RBV-treated viruses, approximately two-thirds of the genome (extending from nucleotide 5255 for MuVi-C or 4122 for ZgA/Cro69 to the genome end) was successfully amplified for preparation of the library.

**Figure 3:**
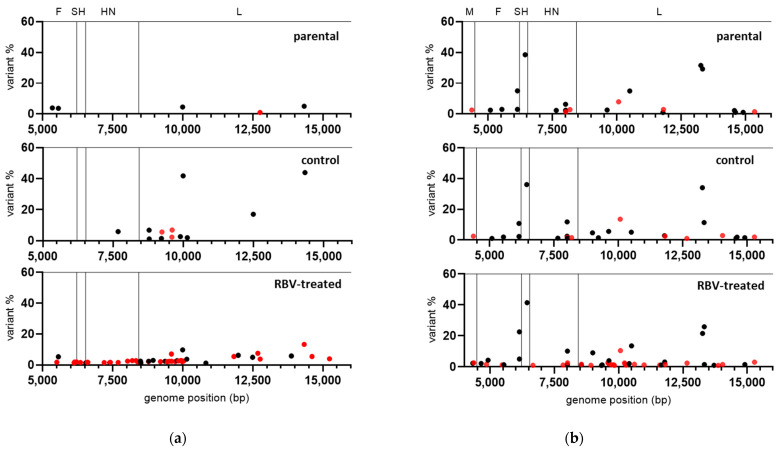
Variant frequency (%) in parental and RBV-treated (250 µM) or control MuVi-C and ZgA/Cro69 viruses from the first passage in Vero and A549 cells, respectively, according to their location in the genome: (**a**) MuVi-C and (**b**) ZgA/Cro69. Corresponding genes are given at the top of the diagrams. RBV-specific mutations (G-to-A and C-to-U) are shown by red dots and all other mutation types by black dots. Only variants present at >1% are shown.

Except for nucleotide change T-to-C at position 6348 of ZgA/Cro69 viruses, which was already present at a high percentage in the parental sample, there were no changes in the consensus sequence of analysed samples. However, data analysis showed an increase in heterogeneity for both RBV-treated viruses in comparison to their respective controls or parental virus (Table 1, Figure 3).

In the case of MuVi-C viruses, 2–4× more heterogenous sites were found in RBV-treated viruses compared to their controls, as well as 1.3–2-fold higher Shannon’s entropy. Variable nucleotide positions were not consistent between same samples from independent experiments with respect to the genomic location; only three positions were variable in both RBV-treated samples, while six of them were variable in both control samples.

For ZgA/Cro69 viruses, all analysed samples (parental, controls, and RBV-treated samples) shared several sites of higher variant frequency, reflecting the heterogenicity present in the parental viral sample (Figure 3). Approximately 45% (in experiment 1) and 60% (in experiment 2) of low-frequency variable sites were found in the RBV-treated samples that were not detected in the parental nor in the control samples. On the other hand, only one heterogeneous position was present in both control samples, which did not occur in the RBV-treated or parental samples.

Next, the distribution of mutation types in our viruses was analysed. The only two mutation types that increased in percentage in RBV-treated viruses compared to their controls were C-to-U (all RBV-treated viruses) and G-to-A (all RBV-treated viruses, except for MuVi-C from experiment 1), although without statistical significance (Table S4). These mutations have been reported as RBV-specific mutations for several other viruses [39,40,41,42] and they are presented together in our data (Table 1, Figure 3).

Together, these data indicate that RBV is able to cause MuV mutagenesis independently of strain or cell type used in our experimental system. The results further support the accumulation of RBV-specific G-to-A and C-to-U transitions.

### 3.4. RBV Concentration Defines MuV Replication Outcome during Several Passages

**Figure 4:**
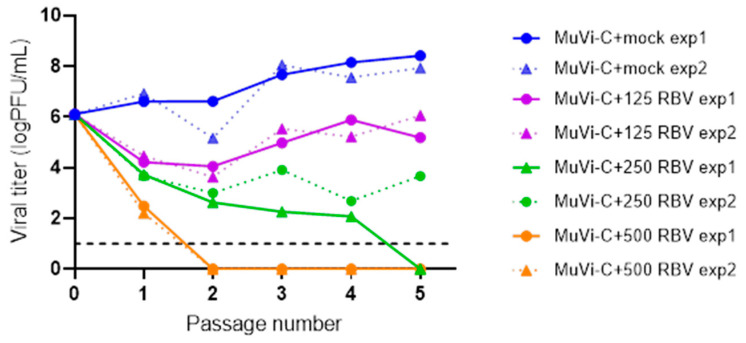
Passaging of MuVi-C in Vero cells treated with different concentrations of RBV. Vero cells were treated with different concentrations of RBV or mock-treated, infected with MuVi-C at MOI 0.001, and then, RBV or medium only were added for an additional 72 h. Titres in supernatants were determined using the plaque assay, and they were further used for subsequent passage at MOI 0.001 in the same conditions. A cutoff value of the plaque assay is shown by a black, dashed line.

To examine if the observed antiviral activity of RBV can lead to virus extinction, we performed consecutive passages of MuVi-C in Vero cells in the absence or presence of 125, 250 or 500 µM RBV (Figure 4). The first two RBV concentrations caused less than a 50% decrease in cell viability, and application of 500 µM RBV resulted in a 54% decrease in cell viability (Figure S2a).

Results obtained from two independent experimental infections show that after the initial drop in virus titre using RBV concentrations of 125 µM, virus growth started to recover in the subsequent passages.

When applying 250 µM RBV in the first experiment, the virus had no detectable titre at passage 5. Still, plenty of plaques were observed when this virus was further passaged in the control medium, while they were not detected if it was passaged in the RBV-supplemented medium (data not shown). Furthermore, virus with no detectable titre was not revived following three additional blind passages in the control medium. On the other hand, the titre of the virus treated with 250 µM RBV in the second experiment initially decreased and then increased again at the fifth passage.

Finally, when using 500 µM of RBV, the virus was extinguished at the second passage and was not revived following an additional three blind passages in the control medium.

These results demonstrate that the outcome of virus replication is strictly conditioned by the RBV concentration applied, and that there are marginal RBV concentrations (250 µM) at which a permanent negative impact on virus fitness is a random event.

### 3.5. Changes in MuV Population Diversity during Passaging in Sublethal Concentration of RBV

To determine the effect of long-term passaging of MuV in the presence of RBV as well as a potential for selection of a resistance phenotype, our MuV strains were sequentially passaged in their respective substrates using either medium without RBV or medium supplemented with 62.5 µM RBV, a concentration that does not drive MuV to extinction.

**Figure 5:**
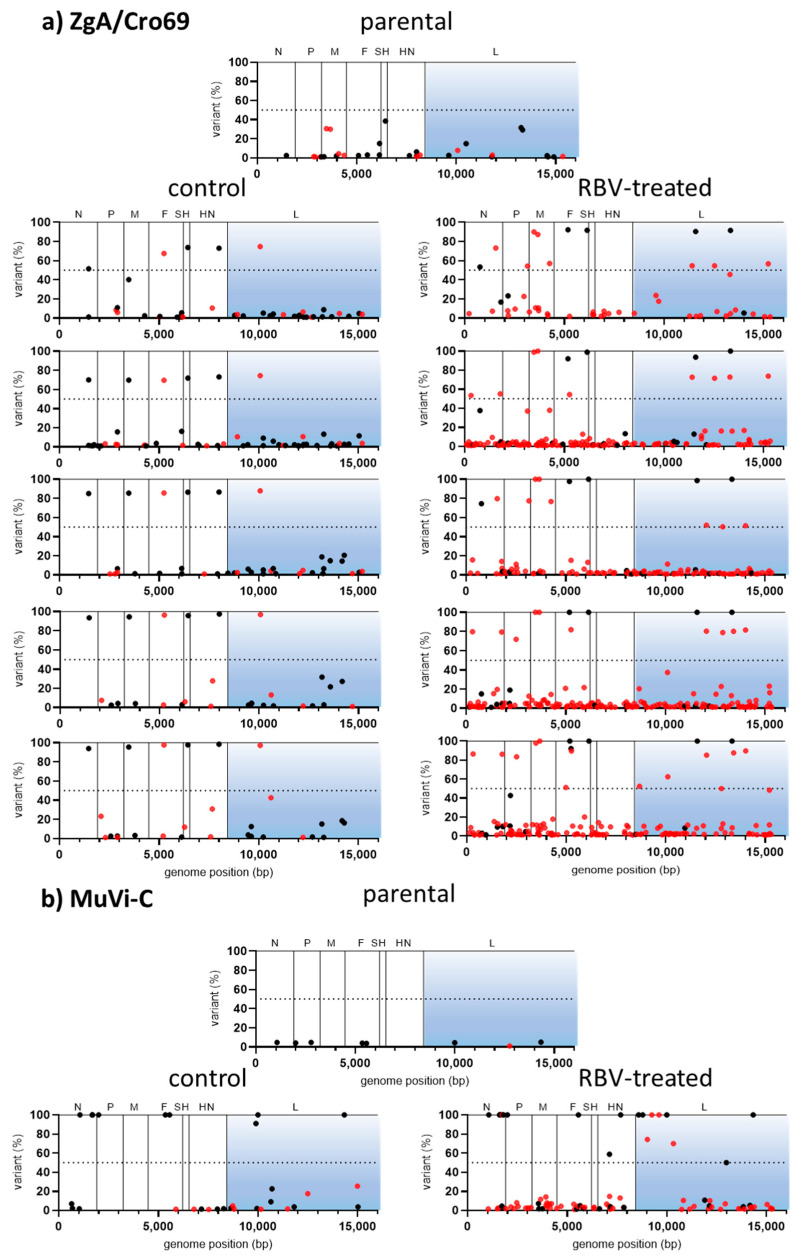
Variant frequency (%) in parental and RBV-treated (62.5 µM) or control (**a**) ZgA/Cro69 and (**b**) MuVi-C viruses from different passages in A549 and Vero cells, respectively, according to their location in the genome. RBV-specific mutations (C-to-U and G-to-A) are shown by red dots and all other mutation types by black dots. Dashed lines indicate a value of 50%; changes present in values higher than 50% are consensus changes. The L gene, where resistance-/fidelity-related mutation/s are expected to emerge, is given in blue. Only variants present in >1% are shown.

Following the initial drop in virus titre of 2.2 log for MuVi-C and 1.3 log for ZgA/Cro69, the difference in titre between control and RBV-treated viruses was maintained throughout the passaging process (Figure S6). Control and RBV-treated viruses from passage eight were then sequenced in their full length. The heterogeneity of both RBV-treated viruses increased compared to respective control viruses from the same passage, as shown by three different diversity indices (Figure 5, Table 2). In the RBV-treated MuVi-C strain, it was more pronounced, being observed in 3× more heterogeneous sites, as well as having a 3.5-fold higher nucleotide diversity and Shannon’s entropy. In the RBV-treated ZgA/Cro69 strain, only a slight increase in the number of variants was observed (1.2× more heterogeneous sites), in addition to 1.9-fold higher nucleotide diversity and a 1.7-fold increase in Shannon’s entropy compared to the control. However, RBV-specific transitions (C-to-U and G-to-A) accumulated at a higher percentage in RBV-treated ZgA/Cro69 virus (84%, X^2^ (1, N = 99) = 23.32, *p* < 0.00001) than in RBV-treated MuVi-C virus (64%, X^2^ (1, N = 127) = 12.6, *p* = 0.000386) compared to their respective controls or parental virus (Table 2 and Table S5). This prompted us to perform additional passages (up to passage 21) of the ZgA/Cro69 strain to elucidate if longer exposure to RBV would result in a decrease in genetic variability and the emergence of RBV resistance.

Control and RBV-treated ZgA/Cro69 viruses from passages 11, 14, 17 and 21 were sequenced in their full length. Contrary to our expectations, data showed that further passaging in RBV-supplemented medium led to an additional increase in heterogeneity resulting in a 4.7-fold higher genetic diversity of RBV-treated virus at passage 21 compared to the control virus (10.64 × 10^−4^ and 2.26 × 10^−4^, respectively (Figure 5, Table 2)). Similarly, the number of RBV-specific mutations (C-to-U and G-to-A) in RBV-treated virus increased with passage number and accounted for 91% of all mutations detected at passage 21 in comparison to only 39% in the control virus ((X^2^ (1, N = 183) = 36.97, *p* < 0.00001), Figure 5, Table 2). Furthermore, genome analysis of RBV-treated virus revealed a total of 17 consensus changes at passage 21 that were not present in the control virus nor in the parental virus (Table S3). Eight consensus changes were located in the L gene; two were nonsynonymous mutations not specific for RBV (11,584 G→T and 13,332 A→T, located in the PRNTase and CD domain, respectively), which were already present in the virus from passage eight, and three were RBV-specific nonsynonymous mutations (12,051 G→A in the PRNTase domain, 12,800 G→A in the CD domain, and 14,018 G→A in the MTase domain). In passaged RBV-treated ZgA/Cro69 virus, there were no changes in the RdRp domain of the L gene nor in the motifs crucial for the polymerase function GDN (in the RdRp domain), HR and GXXT (X denotes any residues in the CRV), and GXGXG (in the CRVI) [43]). In addition to L protein, N and P proteins also form an active RNP complex. In this context, two nonsynonymous RBV-specific changes were detected in the N gene (315 G→A, and 1775 G→A), and one synonymous (2503 C→T) in the P gene.

**Figure 6:**
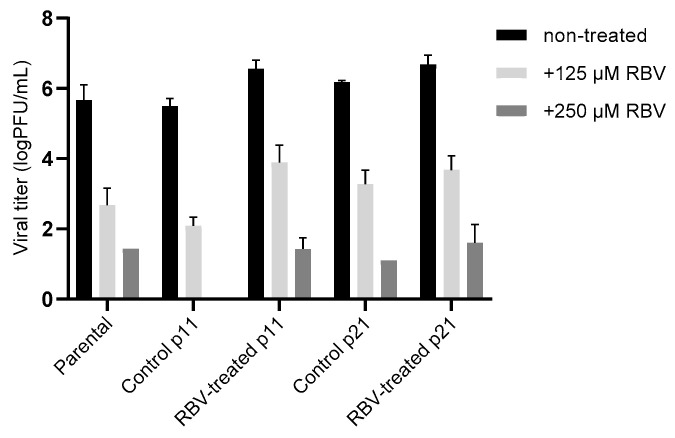
Resistance of passaged RBV-treated and control ZgA/Cro69 viruses to RBV. Parental, control, and RBV-treated viruses from passages 11 and 21 in A549 cells were tested for sensitivity to 125 or 250 µM RBV in A549 cells. Titres in supernatants were determined using the plaque assay. Mean ± standard deviation obtained from 3 biological replicates are shown. Statistical significance between groups was determined using Kruskal–Wallis one-way ANOVA. There were no significant differences between different samples treated with the same RBV concentration.

The resistance of treated and nontreated viral populations to higher concentrations of RBV (125 µM and 250 µM) was further compared in A549 cells. Results obtained for the viruses from the beginning (parental), midphase (passage 11) and end of the passaging process (passage 21) show that a comparable level of virus sensitivity to RBV was maintained throughout the experiment (Figure 6). These results suggest that resistance at a whole-virus population level did not develop in the MuV passaged in 62.5 µM RBV.

## 4. Discussion

There are several experimental conditions that must be fulfilled when setting up an optimal in vitro system for studying viral mutagenesis. Firstly, a combination of viral strain and cell line should facilitate viral replication to a substantial titre, as mutagen effects are subsequently determined through a decrease in viral titre. Secondly, the choice of MOI must not lead to an increase in defective interfering (DI) particles while causing peak titres in infection as early as possible so as to avoid prolonged and detrimental mutagen effects on cells. In the case of MuV, meeting those conditions is challenging because of the slowly replicating nature of the virus.

To estimate if RBV mutagenic activity applies to different MuV strains, we have chosen two viral strains that belong to different genotypes, and despite being extensively passaged in the cell culture, show neurovirulent phenotype (assayed in the conventional animal model for assessment of MuV neurovirulence—a newborn rat model, data not shown). We paired them with cell lines in which they replicate in high titres—Vero and A549. These cell lines have been reported as comparatively resistant to RBV [44,45]. It was further suggested that changing cell state (such as temperature for incubation, passage, or confluence) does not have any effect on RBV antiviral effect. In our experimental setup, these cells showed sensitivity to RBV with respect to cell viability, which decreased upon treatment (IC_50_ dose of 399 µM for Vero cells and 423 µM for A549 cells). It should be noted that the same RBV concentrations had a lower impact on cell viability at earlier time points after infection (data not shown). In agreement with previous observations that RBV has antiproliferative rather than cytotoxic effects on Vero cells [28], we observed no cell cytotoxicity after RBV exposure. Due to the nature of MuV infection with respect to the time needed to obtain efficient cell infection, we opted to use subconfluent instead of confluent cells. Our results regarding RBV’s effect on cell viability are in agreement with those obtained by Shigeta et al. [29] for growing Vero cells (282 µM).

By using selected combinations of MuV strains and cell lines, we have confirmed through dose-dependent experiments that RBV’s antiviral activity is independent of strain and cell type, with ED_50_ even lower than that obtained in similar measurements (37 to 149 µM) [29]. Although we have applied RBV concentrations that result in even more than a 50% decrease in cell viability, we have indirectly shown that reduced cell numbers in infection do not substantially affect viral titre. We therefore concluded that decreases in viral titre following RBV treatment are indeed the result of RBV action. In serial virus passages in the presence of 500 µM RBV, virus titre was not detectable at passage two nor did its growth recover after additional passages in the control medium, suggesting that high RBV doses are able to extinguish virus population.

Leyssen et al. [46] have demonstrated that inhibition of IMPDH is a predominant mechanism by which RBV exerts its antiviral activity against paramyxoviruses, although RBV’s effect on mutational burden in the viral genome was not investigated in their study. Our NGS data showed an increase in MuV population heterogeneity following only one virus passage in the cell culture, which is given by two different diversity indices (Shannon’s entropy and the number of heterogeneous sites). On average, 2.95× more heterogeneous sites and a 1.65-fold higher Shannon’s entropy were shown for one viral strain, and 1.67× more heterogeneous sites and a 1.36-fold higher Shannon’s entropy for another. Importantly, an accumulation of RBV-specific mutations (C-to-U and G-to-A) was demonstrated for both viruses. A lack of insight into the contribution of an increase in heterogeneity and/or RBV-specific mutations to the loss of infectivity seen in passages of MuVs in the presence of 250 or 500 µM RBV, due to inability to sequence a low titre viral samples, is a shortcoming of our study.

Depending on the virus, 5–15 passages are usually necessary to select for a fidelity-related resistant phenotype, which is frequently indicated by titre equalization between viruses passaged in mutagen-containing medium and control medium [45]. The MOI used in our study was 5 to 50 times lower than MOIs previously shown to result in MuV DI emergence [38]. Since the resistant mutant is expected to initially exist in the population at a low frequency, we chose not to further decrease the inoculum population size to prevent the chance of losing these emerging variants. However, contrary to observations made for many other RNA viruses that show the emergence and accumulation of resistant variants following serial passages in sublethal RBV concentrations [14,16,17], we have shown that, in a course of 21 passages with 62.5 µM RBV, MuV does not select for resistance. Deep-sequencing analysis showed that original viral heterogeneity increases with passage number for RBV-treated viruses, while it remains approximately the same for the control viruses (Table 2). The majority of observed substitutions detected in the RBV-treated virus were mutagen-related (C-to-U and G-to-A transitions accounted for > 90% of all observed substitutions).

A 1.5–2-fold difference in genetic diversity has previously been observed between viral populations possessing a natural mutation rate and those with changed replication fidelity for various RNA viruses [13]. In our study, a 4.7-fold higher genetic diversity of RBV-treated virus compared with the control virus was obtained at passage 21 without a change in RBV resistance. The fact that 150 heterogeneous positions were found in RBV-treated virus at the end of the passaging process shows that MuV is able to support high mutational burden within its genome. We consider the observed mutational increase significant, as by using the same sequencing methodology, only up to 50 heterogeneous positions were detected in populations of L-Zagreb vaccine samples from different manufacturing steps (unpublished data). Further investigation is needed to elucidate if the observed increase in heterogeneity is related to the slight drop in virus titre, or dependent on other direct or indirect RBV’s effect on viral life cycle or cell metabolism, or both.

Regarding mutagen resistance, our results do not preclude the possibility that there are resistant variants present at a low level within RBV-treated MuV populations or that their phenotypic effect is masked by the impact of mutations elsewhere in the genome.

In addition, exposing MuV to a higher RBV concentration through serial passages might result in the occurrence of a resistant phenotype. Supporting this is our observation that an RBV concentration of 250 µM led to virus extinction in one experiment (the virus was not recovered following several blind passages in the control medium), but in the second experiment, the virus was recovered after a significant initial drop in titre. Considering these results in combination with results obtained from the consecutive passages in a lower concentration of RBV (62.5 µM), a higher selective pressure is possibly needed to cause mutagen-resistant variant occurrence, which would become dominant in the population. It seems that an RBV concentration of 250 µM is at the boundary of pressure at which MuV can undergo.

This virus–mutagen interplay should be further investigated, as there are no specific, clinically approved treatments for mumps. Except for RBV [28,29,30], MuV antiviral activity has previously been investigated for several compounds of similar chemical structure (nucleoside analogues) including GS-5734 [47], favipiravir [48], 4’-azidocytidine [49], EICAR [46] and TJ13025 [29]. None of these studies have estimated whether the antiviral activity of these compounds involves an increase in mutagenic burden in the MuV genome. Thus, the lethal mutagenesis phenomenon is a promising future research direction for the design or application of mumps antivirals. The application of such antivirals would be beneficial in cases of serious neurological disease consequences such as meningitis or encephalitis [50,51,52,53], which can increase in number in regions without vaccination programs, in cases of large outbreaks, or in individuals who are unvaccinated due to a weakened immunological status.

## 5. Conclusions

The results of this study, obtained by using two combinations of cells and viruses, demonstrate that RBV increases the heterogeneity of viral populations and predominantly induces C-to-U and G-to-A transitions in the MuV genome. Even though MuV is sensitive to RBV, it can readily withstand sublethal concentrations of this mutagen without emergence of a resistance at a whole-virus population level.

## Figures and Tables

**Table 1:** Genetic characterization of RBV-treated (250 µM) and control MuVi-C and ZgA/Cro69 viruses from the first passage in Vero and A549 cells, respectively.

Virus	Treatment (Control or RBV)	Passage	Viral Titre (logPFU/mL)	Total Diversity (×10^−4^)	Shannon’s Entropy (×10^−4^)	Total no. of Heterogeneous Positions	No. of Mutations C-to-U	No. of Mutations G-to-A	% of Mutations C-to-U +G-to-A
	-	0	5.60	0.232	0.225	5	1	0	20
	control 1	1	6.61	0.982	0.855	17	3	1	24
MuVi-C	RBV 1	1	3.93	1.037	1.082	34	15	7	65
	control 2	1	6.93	1.257	0.927	12	2	2	33
	RBV 2	1	3.68	1.962	1.901	47	10	17	57
	-	0	5.34	1.837	1.447	23	5	1	26
	control 1	1	4.96	1.779	1.446	25	5	2	28
ZgA/Cro69	RBV 1	1	3.41	2.177	1.850	40	9	8	43
	control 2	1	5.35	1.804	1.481	28	2	7	32
	RBV 2	1	2.80	2.533	2.151	49	17	9	53

“-” in the Treatment column stands for parental virus; and 1 or 2 stands for independent experiments.

**Table 2:** Genetic characterization of RBV-treated (62.5 µM) and control ZgA/Cro69 and MuVi-C viruses from serial passages in Vero and A549 cells, respectively, based on full-length genome sequences.

Virus	Treatment	Passage no.	Viral Titre (logPFU/mL)	Total Diversity (×10^−4^)	Shannon’s Entropy (×10^−4^)	Total no. of Heterogeneous Positions	No. of Mutations C-to-U	No. of Mutations G-to-A	% of Mutations C-to-U + G-to-A
ZgA/Cro69	-	0	5.34	2.581	2.012	33	8	4	36
	control	8	6.45	3.299	2.617	44	8	7	34
	RBV	8	6.35	6.247	4.561	51	17	26	84
	control	11	8.01	3.918	3.178	56	6	8	25
	RBV	11	6.02	9.596	8.019	158	78	61	86
	control	14	8.56	2.723	2.249	38	3	9	32
	RBV	14	6.31	7.622	6.196	122	55	50	85
	control	17	8.14	2.201	1.783	28	4	6	36
	RBV	17	5.52	10.243	8.747	158	79	67	91
	control	21	7.49	2.260	1.764	28	5	6	39
	RBV	21	6.51	10.639	8.709	150	72	64	91
MuVi-C	-	0	6.11	0.360	0.348	8	1	0	13
	control	8	7.26	1.406	1.165	32	3	6	28
	RBV	8	5.82	4.916	4.140	95	34	27	64

“-” in the Treatment column stands for parental virus.

## Data Availability

Data are contained within the article and supplementary material. Sequencing results were deposited in the NCBI SRA database (https://www.ncbi.nlm.nih.gov/sra, accessed on 16/12/2021) BioProject ID PRJNA776123.

## Supplementary Materials

The following are available online at https://www.mdpi.com/article/10.3390/v13122535/s1, Figure S1. MuV replication in A549 cells; Figure S2. RBV effect on Vero and A549 cells; Figure S3. Dose-dependent antiviral effect of RBV against ZgA/Cro69; Figure S4. A flow chart of performed experiments; Figure S5. Effect of confluence of A549 cells on viral titre; Figure S6. Passaging of MuVi-C and ZgA/Cro69 viruses in Vero and A549 cells, respectively, treated with 62.5 µM RBV; Table S1. List of primers used for amplification of PCR fragments for NGS library preparation; Table S2. NGS data description; Table S3. Changes in the consensus sequence (based on full-length genome sequences) during long-term passaging of RBV-treated (62.5 µM) ZgA/Cro69 virus that are not present as consensus in the controls nor in the parental virus; Table S4. Percentage of each mutation type occurring in control and RBV-treated (250 µM) MuVi-C and ZgA/Cro69 viruses from the first passage; Table S5. Percentage of each mutation type occurring in control and RBV-treated (62.5 µM) MuVi-C and ZgA/Cro69 viruses from passages 8 and 21.
