# Supplementary material for: Influence of Ribavirin on Mumps Virus Population Diversity
Source: Viruses. 2021 Dec 17;13(12):2535. doi: 10.3390/v13122535 (PMC8706638; doi:10.3390/v13122535)
Supplement: Supplementary file 1 [file viruses-13-02535-s001.zip › viruses-1465189-supplementary.pdf]

Figure S1. MuV replication in A549 cells. A549 cells layers were infected with ZgA/Cro69 at MOI 0.01, supernatants were collected from day 1 to day 6 post infection and titres were determined using plaque assay.

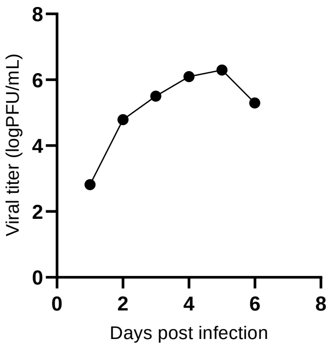

Figure S2. RBV effect on Vero and A549 cells: (**a and b**) Cells were seeded in 96-well format and treated with increasing concentrations of RBV (3.9-1000 µM) for 72 or 96 hours for Vero (a) and A549 cells (b), respectively, and its influence was assayed using MTT assay. Percentage of cell viability (y axis) is expressed relative to untreated cells. Each point represents mean ± standard deviation derived from 2 independent experiments with 6 biological replicates each (n=12). Significant differences among multiple groups were determined using one-way ANOVA and post-hoc Tukey Kramer test; non-significant values are marked by "ns", all other values are statistically significant ($p<0.05$); (**c and d**) Microscopical images (magnification 400x) of Vero (c) and A549 (d) cells following RBV treatment.

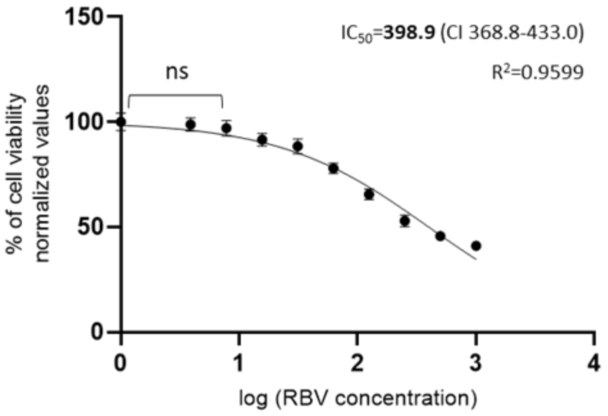

(a)

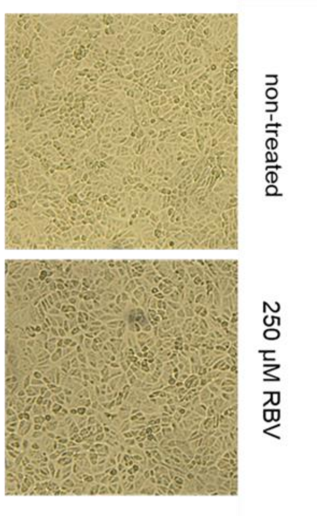

(c)

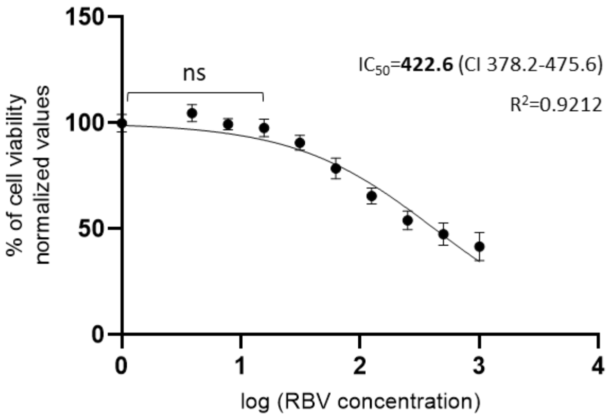

(b)

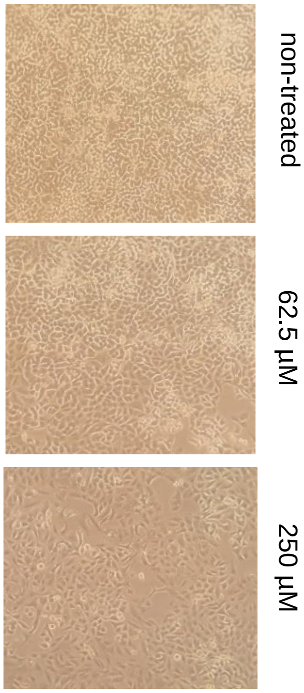

(d)

Figure S3. Dose-dependent antiviral effect of RBV against ZgA/Cro69. A549 cells were treated with different concentrations of RBV or mock-treated, infected with ZgA/Cro69 at MOI 0.01, and then media with or without RBV was added for additional 96 hours. Titres in supernatants were determined using the plaque assay; titres (logPFU/mL) are ploted on the right y axis, and the titre (number of plaques) of virus obtained from RBV-treated cells as a percentage of the virus titre obtained from untreated, control cells is given on the left y axis. Mean ± standard deviations are shown from 3 biological replicates.

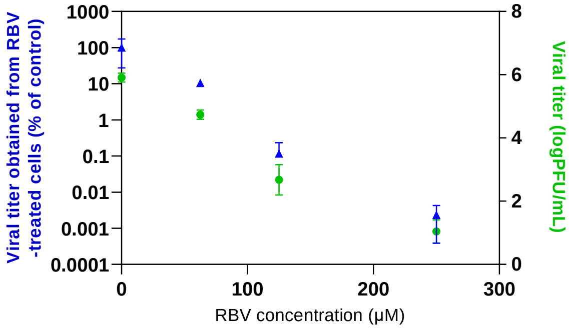

Figure S4. A flow chart of performed experiments. Questions that each of the experiments needed to answer are indicated in ovals in chronological order. On top of each bracket that represents description of experimental work, concentrations of RBV used are pointed out. Below brackets are indicated samples that were sequenced using NGS method.

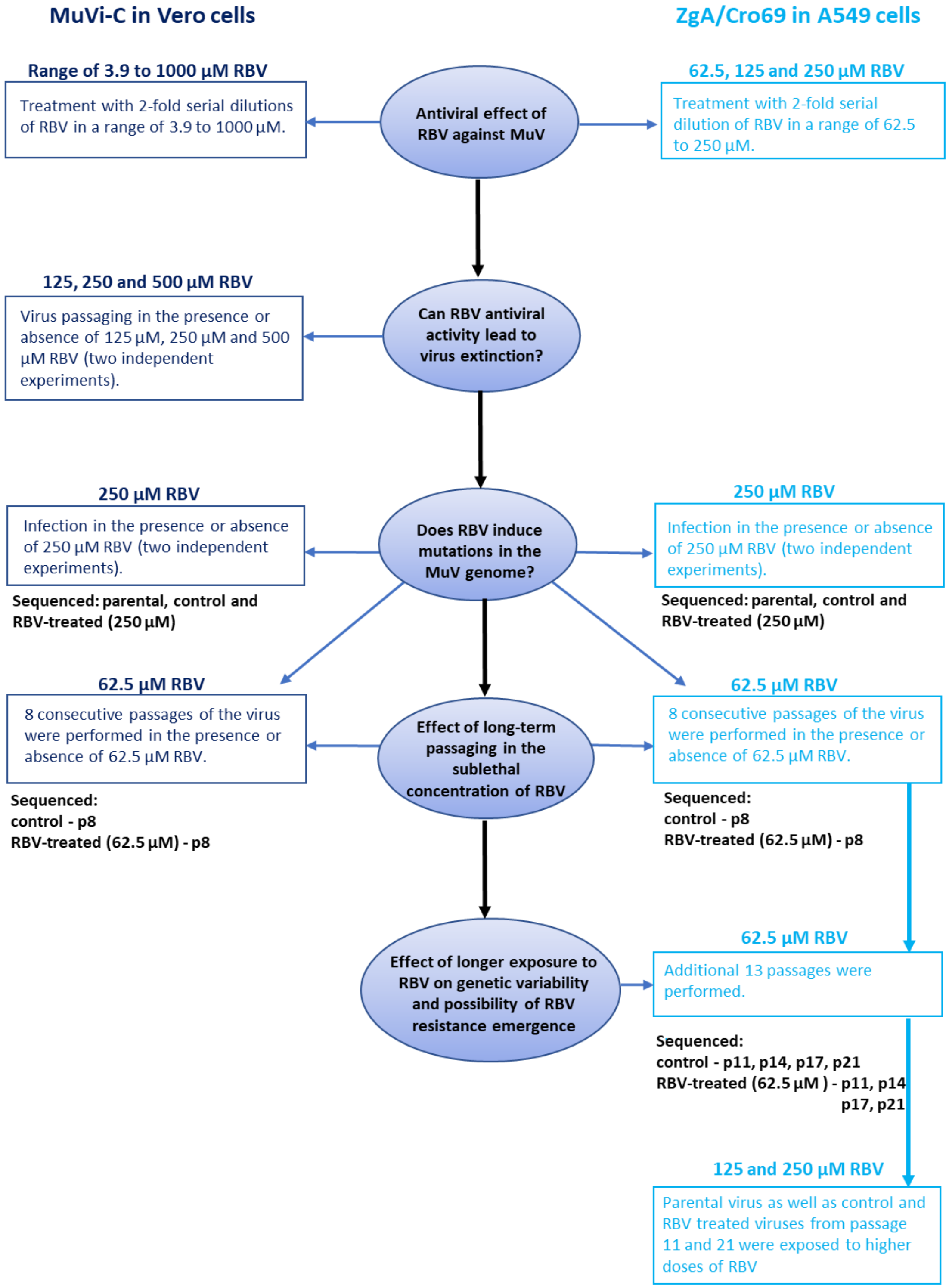

Figure S5. Effect of confluence of A549 cells on viral titre. A549 cells were seeded to obtain 50%, 75% and 100% confluence on the day of infection, infected with ZgA/Cro69 at MOI 0.01, supernatants were collected after 96 hours and titres in supernatants were determined using plaque assay. Statistical significance among groups was determined using Kruskall-Wallis One-Way ANOVA (ns, non-significant).

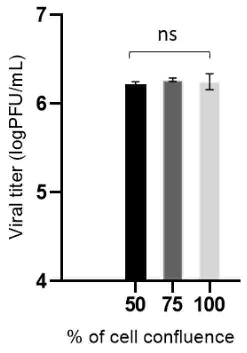

Figure S6. Passaging of MuVi-C and ZgA/Cro69 viruses in Vero and A549 cells, respectively, treated with 62.5 µM RBV. Cells were treated with RBV-supplemented medium or mock-treated, infected with MuVi-C at MOI 0.001 or ZgA/Cro69 at MOI 0.01, and then RBV-supplemented medium or control medium were added for additional 72 or 96 hours, respectively. Titres in supernatants were determined using plaque assay and supernatants were used for subsequent passage at same MOI.

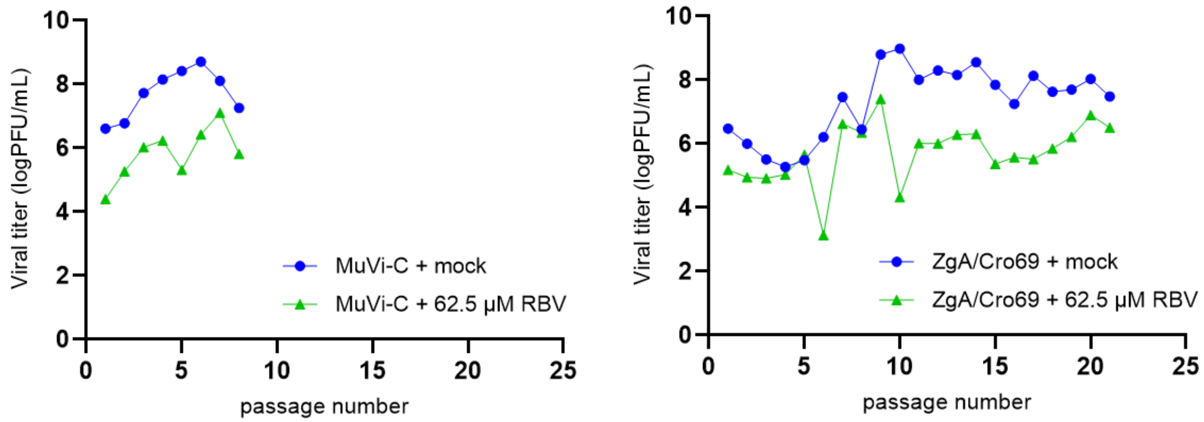

Table S1. List of primers used for amplification of PCR fragments for NGS library preparation. Different combination of primers was used for different viral strains. Amplicon size varied from approximately 700 bp to 5500 bp.

Primer code*	Primer sequence (5'-3')	Genome binding position (bp)
N0_nextera(+)	TGCTGACTGCTCTGATCGTGGTAGCTGATAACCGTCAGTGTGATGTGATAGACCAAGGGGARAAAGAAGATGGGATATTG	1-29
Deopti g2(+)	ATTTCGAGGCCGGGCTCGATCCTCACCTT	79-107
Deopti g4(+)	CAGGACAATCTAGCCACAGCTAACTGCCC	1836-1864
P10rev(-)	TTGTGGCCTTGCTTAACGAG	2077-2096
M5(+)	TAGTCCATGCAGGCGGTCAC	4102-4121
P START, HindIII(+)	ATTATTAAGCTTATGGATCAATTTATAAAACA	4157-4176
F2(-)	AGCTGGTTATCAAGGATCT	5102-5120
F10(+)	GGCATTGTCACCGATTAGTA	5187-5206
F15(+)	GTATGACGCCTGCAGTGGTTC	5234-5254
MR4ms(-)	GCTCGAAATTGAGTAGAAGT	5408-5427
F8(-)	CCAAGATCCTGTCTGGCAAT	5457-5476
LZ9(+)	TTCTCTATCGGCCATCCACT	7082-7101
LZ13(+)	TTGTCTGTGCCTGGAATCAG	7752-7771
LZ6(-)	GCTCGCAATTTGTAACTAGG	7774-7793
LZ14(-)	AGAGGAGTTCATACGGCCAC	7891-7910
L9(+)	AAGGCTAATGCGAAGCACTC	10550-10568
L26(-)	ATTCAGAGCTGTATCAAGGTCA	10615-10636
L8(-)	CCTCCACGAGGAGAAACTA	10650-10668
MR9ms(+)	AGAAGGAGCAAGCTTATAAA	10830-10849
L12(-)	GTCTCACCTCCAGTGAATACC	11715-11735
L23(-)	CTGATGATTGGCCCTTTAGGA	14760-14780
L24(+)	GGACTCCAAGCACAAGAA	14649-14666
L25_nextera(-)	TGGTCATAATGCTGACTGCTGTGATCGTGGTAGTAGATTAGGGTCAGTGTGATGTACCAAGGGGAGAAAGTAAAATCAAT	15360-15384

*(+) or (-) stands for forward or reverse primer orientation, respectively.

Table S2. NGS data description.

Sample	No. of reads	No. of reads after QC	% of mapped reads	No. of reads after removal of reads with more than 2 mismatches to reference	Mean coverage	Mean coverage St. Dev.
MuVi-C parental	470.590	326.368	99.5	323.441	2.637	819
MuVi-C control p1, exp 1	791.188	641.322	98.6	628.967	4.655	4.567
MuVi-C RBV-treated p1, exp 1	1.057.484	864.258	95.1	816.756	6.075	4.875
MuVi-C control p1, exp 2	1.439.204	1.197.064	99.4	1.183.387	8.930	10.065
MuVi-C RBV-treated p1, exp 2	4.654.772	3.957.698	97.1	3.821.604	29.287	16.693
MuVi-C control p8, exp 1	633.024	400.156	98.2	391.035	3.132	2.296
MuVi-C RBV-treated p8, exp 1	630.754	357.894	95.1	338.050	2.650	5.587
ZgA/Cro69 parental	2.835.066	1.074.502	96.0	1.017.702	7.559	2.378
ZgA/Cro69 RBV-treated p1, exp 1	880.938	589.464	98.6	578.628	4.675	4.468
ZgA/Cro69 control p1, exp 1	671.860	426.100	98.6	418.204	3.354	2.458
ZgA/Cro69 RBV-treated p1, exp 2	564.518	340.510	98.0	3.310.943	2.643	2.466
ZgA/Cro69 control p1, exp 2	564.172	348.572	98.7	341.971	2.736	2.386
ZgA/Cro69 control p8/1, exp 1	1.297.784	556.594	94.5	516.801	3.911	10.219
ZgA/Cro69 control p8/2, exp 1	1.951.242	825.126	93.6	758.083	5.733	18.737
ZgA/Cro69 RBV-treated p8/1, exp 1	438.630	173.972	93.9	159.405	1.193	3.337
ZgA/Cro69 RBV-treated p8/2, exp 1	1.295.064	513.464	93.4	468.522	3.498	12.113
ZgA/Cro69 control p11/1, exp 1	2.524.084	914.508	95.9	865.583	6.392	2.432
ZgA/Cro69 RBV-treated p11/1, exp 1	2.924.456	1.106.944	95.8	1.047.245	7.769	3.489
ZgA/Cro69 control p14/1, exp 1	2.762.406	1.115.266	96.8	1.069.556	8.021	4.602
ZgA/Cro69 RBV-treated p14/1, exp 1	2.625.140	972.934	95.6	919.329	6.813	2.061
ZgA/Cro69 control p17/1, exp 1	1.656.620	695.950	93.8	641.171	4.834	13.815
ZgA/Cro69 control p17/2, exp 1	1.065.928	410.048	93.6	374.786	2.794	8.264
ZgA/Cro69 RBV-treated p17/1, exp 1	1.619.980	638.200	96.2	599.177	4.482	6.836
ZgA/Cro69 RBV-treated p17/2, exp 1	1.423.842	535.614	96.8	504.179	3.744	4.189

ZgA/Cro69 control p21/1, exp 1	1.232.634	529.144	97.1	504.498	3.821	4.005
ZgA/Cro69 control p21/2, exp 1	3.224.044	1.447.894	96.5	1.375.540	10.453	12.337
ZgA/Cro69 RBV-treated p21/1, exp 1	1.546.698	634.712	92.8	576.148	4.337	14.914
ZgA/Cro69 RBV-treated p21/2, exp 1	1.458.256	596.106	95.4	556.035	4.180	7.578

p, passage
exp, experiment
/1 or /2, samples sequenced in duplicate
QC, quality control

Table S3. Changes in the consensus sequence (based on full length genome sequences) during long-term passaging of RBV-treated (62.5 μM) ZgA/Cro69 virus that are not present as consensus in the controls nor in the parental virus.

Nucleotide change	Amino acid change	Position in the genome (gene)	Parental p0	Control p8	RBV-treated p8	Control p11	RBV-treated p11	Control p14	RBV-treated p14	Control p17	RBV-treated p17	Control p21	RBV-treated p21
G --> A	Arg --> Lys	315 (N)	-	-	-	-	+ 53%	-	- 16%	-	+ 80%	-	+ 87%
G --> A	Val --> Met	1775 (N)	-	-	-	-	+ 55%	-	- 14%	-	+ 80%	-	+ 87%
C --> T	-	2503 (P)	-	-	-	-	- 41%	-	- 11%	-	+ 72%	-	+ 94%
C --> T	Pro --> Ser	3474 (M)	- 30%	-	+ 90%	-	+ 99%	-	+ 100%	-	+ 100%	-	+ 98%
C --> T	Pro --> Leu	3670 (M)	- 30%	-	+ 87%	-	+ 100%	-	+ 100%	-	+ 100%	-	+ 100%
G --> A	-	4981 (F)	-	-	-	-	-	-	-	-	- 21%	-	+ 51%
A --> G	-	5178 (F)	-	-	+ 92%	-	+ 92%	-	+ 98%	-	+ 100%	-	+ 100%
C --> T	Thr --> Ile	5261 (F)	-	-	- 2%	-	+ 54%	-	- 15%	-	+ 82%	-	+ 90%
T --> A	Ser --> Arg	6138 (F)	- 15%	-	+ 92%	-	+ 99%	-	+ 100%	-	+ 100%	-	+ 100%
G --> A	-	10102 (L)	-	-	-	-	-	-	- 11%	-	- 37%	-	+ 62%
G -->T	Glu --> Asp	11584 (L)	-	-	+ 90%	-	+ 94%	-	+ 99%	-	+ 100%	-	+ 100%
G --> A	Gly --> Glu	12051 (L)	-	-	-	-	- 16%	-	+ 52%	-	+ 80%	-	+ 85%
G --> A	Val --> Ile	12800 (L)	-	-	-	-	-	-	-	-	- 23%	-	+ 50%
C --> T	-	12874 (L)	-	-	-	-	- 16%	-	+ 50%	-	+ 79%	-	+ 13%
A --> T	Tyr --> Phe	13332 (L)	- 29%	-	+ 92%	-	+ 100%	-	+ 100%	-	+ 100%	-	+ 100%
G --> A	-	13417 (L)	-	-	-	-	- 16%	-	+ 48%	-	+ 80%	-	+ 88%
G --> A	Val --> Ile	14018 (L)	-	-	-	-	- 17%	-	+ 51%	-	+ 82%	-	+ 90%

Nucleotide changes are given as appearing in the sequenced DNA; for example, G-to-A corresponds to C-to-U, and C-to-T to G-to-A in the viral RNA genome.
"-" indicates that nucleotide change is not present in viral population in more than 50%; if the changed nucleotide is present at frequency >1%, the percentage is indicated.

Table S4. Percentage of each mutation type occurring in control and RBV-treated (250 µM) MuVi-C and ZgA/Cro69 viruses from the first passage.

		**MuVi-C**				**ZgA/Cro69**			
		C1	R1	C2	R2	C1	R1	C2	R2
**transitions**	**C --> U**	**18**	**45**	**17**	**21**	**25**	**40**	**20**	**23**
	**G --> A**	**6**	**21**	**17**	**36**	**7**	**2**	**8**	**18**
	**U --> C**	12	6	8	17	18	21	20	18
	**A --> G**	6	3	8	2	18	19	20	18
**transversions**	**U --> G**	0	3	0	4	7	2	8	0
	**U --> A**	18	9	17	4	7	7	8	5
	**G --> U**	24	3	0	2	4	2	4	3
	**G --> C**	0	6	0	4	0	0	0	0
	**A --> U**	0	0	8	2	7	2	8	5
	**A --> C**	0	0	8	0	4	2	4	8
	**C --> G**	0	3	0	0	0	0	0	0
	**C --> A**	18	0	17	6	4	0	0	5

C, control virus

R, RBV-treated virus

1 or 2, results from individual experiments

Table S5. Percentage of each mutation type occurring in control and RBV-treated (62.5 µM) MuVi-C and ZgA/Cro69 viruses from the passages 8 and 21.

		**MuVi-C (passage 8)**		**ZgA/Cro69 (passage 8)**		**ZgA/Cro69 (passage 21)**	
		**C**	**R**	**C**	**R**	**C**	**R**
**transitions**	**C --> U**	**9**	**36**	**18**	**31**	**18**	**46**
	**G --> A**	**19**	**28**	**16**	**51**	**21**	**42**
	**U --> C**	9	11	14	2	11	1
	**A --> G**	25	14	27	4	25	3
**transversions**	**U --> G**	0	1	0	2	0	1
	**U --> A**	6	3	5	2	4	1
	**G --> U**	6	3	5	4	11	3
	**G --> C**	0	0	2	0	4	0
	**A --> U**	6	2	2	2	0	1
	**A --> C**	9	0	5	0	4	0
	**C --> G**	0	0	0	0	0	1
	**C --> A**	9	2	7	4	4	2

C, control virus

R, RBV-treated virus
